# The Newly Developed Elderly Nutrient-Rich Food Score Is a Useful Tool to Assess Nutrient Density in European Older Adults

**DOI:** 10.3389/fnut.2019.00119

**Published:** 2019-08-02

**Authors:** Agnes A. M. Berendsen, Charlotte S. Kramer, Lisette C. P. G. M. de Groot

**Affiliations:** Division of Human Nutrition & Health, Wageningen University & Research, Wageningen, Netherlands

**Keywords:** nutrient density, diet, elderly, nutrient profiling, nutrient-rich food index, diet quality

## Abstract

**Objective:** To develop a nutrient-rich food (NRF) score that captures dietary reference values for older adults and to validate this against a diet index that was specifically designed to assess adherence to dietary guidelines for the older population.

**Design:** A cross-sectional study within the Dutch National Food Consumption Survey (DNFCS, *n* = 735 men and women aged 70–94 years, enrolled between October 2010 and February 2012) and within the NU-AGE study (*n* = 250 men and women aged 65–79 years, enrolled between April 2012 and March 2013). Dietary intake was assessed by means of two non-consecutive dietary record assisted 24-h recalls and 7-day food records, respectively. Structured questionnaires collected data on lifestyle and socio-economic information. Anthropometrics were measured by trained dieticians or research assistants. We evaluated Elderly NRF (E-NRF) scores against the NU-AGE index, a measure of adherence to European dietary guidelines for the aging population. The E-NRF scores were composed of nutrients that: (1) have been shown to be of inadequate intake in the aging population (>20%), (2) were defined as nutrients of public health relevance, and (3) were associated with relevant health outcomes.

**Results:** The E-NRF score that best predicted the NU-AGE index included seven nutrients to encourage (protein, dietary fiber, folate, vitamin D, calcium, magnesium, potassium) and three nutrients to limit (saturated fat, sodium and mono- and disaccharides) on a 100-kcal basis, the E-NRF7.3 score (model R^2^ 0.27 in DNFCS and 0.41 in NU-AGE). Food groups contributing the most to the individual E-NRF7.3 scores were vegetables, bread, potatoes and milk and milk products.

**Conclusion:** The E-NRF7.3 score is a useful tool for assessing nutrient density of diets within the older population. No index has previously been developed with the aim of evaluating nutrient density of diets and foods specifically capturing dietary reference values for older adults.

## Introduction

Changing demographics in Europe result in an increasing proportion of older people; the number of people aged 65 years and over is projected to rise from 97.7 million (19.2%) in 2016 to 151 million (29.1%) in 2080 (1). Longer lives are often accompanied by increased morbidity and suboptimal health. Suboptimal health in people aged 60 years and over comprises 35% of the burden of disease in high-income countries, which is mainly attributable to degenerative diseases, such as cardiovascular disease and cancer (2).

Many degenerative diseases are influenced by inadequate nutrient intakes such as low protein, fiber, and micronutrient intakes on one hand (3–5) and excess intakes of glucose and fat causing low-grade inflammation on the other hand (6, 7). Besides degenerative diseases, low micronutrient intakes among older adults are associated with adverse outcomes such as increased fracture risk for low vitamin D intake (8, 9). Moreover, increasing numbers of nutrient deficiencies within individuals are associated with a higher risk of frailty (10).

Inadequate nutrient intakes are common, as observed in recent studies. A systematic review on macronutrient intakes in community-dwelling elderly from 46 studies showed inadequate protein intakes of 10 and 12% when using an Average Requirement (AR) of 0.66 g/kg bodyweight/day, rising to 27 and 23% in men and women, respectively when applying more recent suggestions of an AR of 0.83 g/kg bodyweight/day (11). Additionally, dietary fiber intakes were below recommendations in most European countries according to a recent review on nutrient intakes from 18 European National Nutrition Surveys (12). A recent systematic review of 37 studies from Western countries including participants aged 65 years and over observed nutrient intake inadequacies that were of public health concern with over 30% reporting an intake below the AR for six nutrients, namely vitamin D, vitamin B1, vitamin B2, calcium, magnesium and selenium (13). Additionally, over 20% showed inadequate intakes for folate, vitamin B6, and vitamins A, C and E (14). This partly matches nutrients identified by EURRECA as the most relevant for public health of elderly people at European level, namely vitamin D, vitamin B12, folate, calcium and iron (15). In the most recently published data on nutrient intakes in the Netherlands including men and women aged 70–94 years of age, 95% of men and 91% of women had inadequate vitamin D intakes, whereas potentially inadequate intakes were observed for vitamin A, B2, B6, folate equivalents, selenium and (for men) vitamin C (16). Another study in the Netherlands (252 men and women aged 65–79 years of age), observed higher percentages of inadequate intakes for vitamin D (98%), selenium (41%) and vitamin B6 (54%), even when taking into account the use of supplements (87, 36, and 20%, respectively) (17). Additionally, omega-3 fatty acids were identified as important nutrients for healthy aging (18). Intakes of sodium, saturated fat and free sugar on the other hand have been reported to be much higher than the current recommendations (12). While energy requirements decrease with age (19), nutrient requirements stabilize or increase, contributing to inadequate nutrient intakes in older adults and necessitating a more nutrient-dense diet (20).

Nutrient density can be expressed by composite indices of nutritional quality. These nutrient density scores reflect the nutrient density of a food or diet in relation to dietary reference values per standard unit (e.g., per 100 gram or 100 kcal). Calculating nutrient content per standard unit, instead of using food groups in dietary indices, means nutrient density can be calculated. This allows for simple calculations, both on the food level as well as the diet level, of how to increase nutrient intake while keeping energy intakes stable. Additionally, nutrient density scores are widely applicable, because they do not rely on specific foods and food groups of which the intake varies between regions. Moreover, nutrient density scores can be used to calculate which foods have maximum nutrient-to-kcal ratios for the lowest price (21). The NRF9.3 score includes nine nutrients to encourage and three nutrients to limit based on nutrients of concern for American adults (22). The NRF9.3 has been validated with the Healthy Eating Index in the USA (23) and with the Dutch Healthy Diet index (DHD) in the Netherlands (24). The NRF9.3 can be used to study associations with health outcomes, such as cardiovascular diseases and mortality (25) and to study the contribution of foods or food groups to the overall nutrient intake (17, 24). However, as the NRF9.3 is based on requirements for American adults it might be of limited use for studying nutrient density within an aging European population as it does not include several important nutrients for older adults, such as vitamin D and folate (17). To our knowledge, there is no nutrient rich food score that specifically captures relevant nutrients for older European adults. Such nutrient density score for older adults could be used to support nutrition and health claims (26), help older people to identify nutrient-rich foods and shape their food purchase decisions by which their diet quality could improve (27).

The aim of the present study was to develop a nutrient-rich food score (Elderly-NRF) that may be used as a tool to distinguish high and low nutrient-dense diets by targeting dietary reference values for the older population. Additionally, we evaluated the E-NRF score against a diet score which was specifically developed to measure adherence to dietary guidelines for older adults (NU-AGE index).

## Methods

### Study Design and Population

The E-NRF scores were developed and evaluated within the Dutch National Food Consumption Survey (DNFCS) 2010–2012 (16) and within the NU-AGE study (28).

The DNFCS was conducted in non-institutionalized elderly aged 70–94 years in the Netherlands. In total, 3,138 individuals were invited, of which 2,848 were eligible and 739 agreed to participate. For the present study, 735 participants aged 70–94 years (369 men and 366 women) were included, after excluding participants with unlikely energy intakes (<500 or >3,500 kcal; *n* = 4).

The NU-AGE study is a 1-year, randomized, parallel trial with the aim to investigate whether a newly designed, personally tailored Mediterranean-like dietary pattern, targeting dietary recommendations for people over 65 years of age (NU-AGE diet) can counteract or slow down the inflammageing process. The study was carried out in five European study centers. For the present study we used baseline data of the Dutch cohort including 252 apparently healthy men and women aged 65–79 years enrolled between April 2012 and March 2013. The rationale and design of this intervention study are described in detail elsewhere (28, 29). Ethical approval was provided by the Wageningen University Medical Ethics Committee (The Netherlands). The trial is registered at clinicaltrials.gov (NCT01754012). For the present study, 250 participants were included, after excluding participants with missing data on supplement use (*n* = 1) and unlikely energy intakes (<500 or >3,500 kcal; *n* = 1).

All study procedures were in accordance with the ethical standards of the Helsinki Declaration. All participants gave written informed consent before participating.

### Dietary Intake and Covariate Assessment

Within the DNFCS, dietary intake data were collected by means of two non-consecutive dietary record assisted 24-h dietary recalls from October 2010 to February 2012. Each individual was interviewed twice with an interval of 2–6weeks. The recalls were spread equally over all days of the week and seasons. The two 24-h dietary recalls were conducted during home visits using the computer-directed interview program EPIC-SOFT (30, 31). During these visits the dietary records were checked for incompleteness and for the use of household measures to indicate consumption amounts at home. Consumption data were linked to the 2011 Dutch food composition database (Nederlands voedingsstoffenbestand, NEVO) (32) and averaged over 2 days. Foods were organized into twenty-three food groups by the NEVO classification. A general questionnaire assessed demographics, health and lifestyle factors and dietary supplement use. Highly educated people were defined as having higher vocational education or university. Physically active was defined as a minimum of 30 min of moderately intense activity ≥5 days a week. Weight and height were assessed at the participants home. All intake data collection and anthropometric measurements were carried out by trained dieticians (16).

Within the NU-AGE study, food records on seven consecutive days were used to assess dietary intake. To remind participants to record all foods consumed, a preformatted food record was used including eight meal occasions referring to the current day. In advance, participants had a face-to-face training and received written instructions to keep complete and accurate food records (33, 34). Portion sizes were reported in national household measures, based on pictures or measured in gram or milliliters. During a 1-h interview with a trained dietician/research nutritionist the food record was reviewed and checked for frequently used household measures to ensure an adequate level of detail in describing foods and food preparation methods (34). Consumed foods were coded according to standardized coding procedures. Subsequently, each ingredient or food was translated into nutrients and converted into twenty-three food groups by the Dutch food composition database (NEVO) 2011 (32). Data on supplement use was obtained by means of a self-reported supplement questionnaire and checked by a trained dietician/research nutritionist. Participants completed questionnaires about their health and lifestyle. Education was assessed as years of full-time education (>16 years of education, equivalent to a bachelor degree, was considered as highly educated). Physical activity was assessed by means of the Physical Activity Scale for Elderly (PASE). Anthropometric measurements were done by trained research assistants at the research center.

### Development and Calculation of Elderly NRF (E-NRF)

Table 1 gives an overview of the stepwise development of the Elderly-NRF (E-NRF) scores. These scores were based upon a selection of nutrients. All positive models contained protein and dietary fiber since inadequate intakes are common and adequate intakes are associated with disease prevention (11, 12, 35, 36). Furthermore, nutrients were selected if they were both shown to be of inadequate intake (≥20%) as reported in a recent review from ter Borg et al. and if they were defined as nutrients of high public health relevance for elderly by EURRECA (14, 15), resulting in the selection of vitamin D, folate and calcium. Additionally, micronutrients that were associated with a health outcome relevant to elderly according to EURRECA, including magnesium, iron, selenium, iodine, potassium, zinc, and vitamin B6, vitamin B12, vitamin C, vitamin E, and vitamin K (15), were selected and individually added to the models. Lastly, polyunsaturated fatty acids (PUFA) were selected, as they are related to health, according to the latest systematic review on nutrients and aging (18). Eventually, the positive scores included protein, dietary fiber, PUFA and a range of micronutrients, namely vitamin B6, vitamin B12, vitamin C, vitamin D, vitamin E, folate, calcium, magnesium, potassium, zinc, iodine, iron, copper, and selenium; the negative scores comprised saturated fat, sodium and total mono- and disaccharides.

**Table 1:** Elderly nutrient-rich food scores; models with nutrients to encourage, nutrients to limit, and full models including both.

**E-NRF model**	**Nutrient-rich components**			**Nutrients to limit**
	**Macronutrients**	**Vitamins**	**Minerals**	
LIM3				Saturated fat, total mono- and disaccharides, Na
E-NR5	Protein, dietary fiber	D, folate	Ca	
E-NR6Zn	Protein, dietary fiber	D, folate	Ca, Zn	
E-NR6I	Protein, dietary fiber	D, folate	Ca, I	
E-NR6VitE	Protein, dietary fiber	D, folate, E	Ca,	
E-NR6VitC	Protein, dietary fiber	D, folate, C	Ca	
E-NR6Se	Protein, dietary fiber	D, folate	Ca, Se	
E-NR6B12	Protein, dietary fiber	D, folate, B12	Ca	
E-NR6B6	Protein, dietary fiber	D, folate, B6	Ca	
E-NR6Fe	Protein, dietary fiber	D, folate	Ca, Fe	
E-NR6Mg	Protein, dietary fiber	D, folate	Ca, Mg	
E-NR6K	Protein, dietary fiber	D, folate	Ca, K	
E-NR7	Protein, dietary fiber	D, folate	Ca, K, Mg	
E-NR8	Protein, dietary fiber, PUFA	D, folate	Ca, K, Mg	
**FULL MODELS**				
E-NRF5.3	Protein, dietary fiber	D, folate	Ca	Saturated fat, total mono- and disaccharides, Na
E-NRF6.3-Zn	Protein, dietary fiber	D, folate	Ca, Zn	Saturated fat, total mono- and disaccharides, Na
E-NRF6.3-I	Protein, dietary fiber	D, folate	Ca, I	Saturated fat, total mono- and disaccharides, Na
E-NRF6.3-VitE	Protein, dietary fiber	D, folate, E	Ca,	Saturated fat, total mono- and disaccharides, Na
E-NRF6.3-VitC	Protein, dietary fiber	D, folate, C	Ca	Saturated fat, total mono- and disaccharides, Na
E-NRF6.3-Se	Protein, dietary fiber	D, folate	Ca, Se	Saturated fat, total mono- and disaccharides, Na
E-NRF6.3-B12	Protein, dietary fiber	D, folate, B12	Ca	Saturated fat, total mono- and disaccharides, Na
E-NRF6.3-B6	Protein, dietary fiber	D, folate, B6	Ca	Saturated fat, total mono- and disaccharides, Na
E-NRF6.3-Fe	Protein, dietary fiber	D, folate	Ca, Fe	Saturated fat, total mono- and disaccharides, Na
E-NRF6.3-Mg	Protein, dietary fiber	D, folate	Ca, Mg	Saturated fat, total mono- and disaccharides, Na
E-NRF6.3-K	Protein, dietary fiber	D, folate	Ca, K	Saturated fat, total mono- and disaccharides, Na
E-NRF7.3	Protein, dietary fiber	D, folate	Ca, K, Mg	Saturated fat, total mono- and disaccharides, Na
E-NRF8.3	Protein, dietary fiber, PUFA	D, folate	Ca, K, Mg	Saturated fat, total mono- and disaccharides, Na

*NR, nutrients to encourage; LIM, nutrients to limit; E-NRF, full elderly nutrient-rich food score*.

Population Reference Intakes and Adequate Intakes as set by the European Food Safety Authority (37–50), the Nordic Council of Ministers (51), the Health Council of the Netherlands (52) as well as the labeling reference intake values as set by the European Food Safety Authority (53) were used as Dietary Reference Values (DRV) (Table 2). The percentage of DRV for each nutrient was capped at 100% DRV to avoid overvaluing food items that provide very large amounts of a single nutrient, such as fortified foods (22).

**Table 2:** Dietary reference values for selected nutrients used in developing the E-NRF7.3.

**Nutrient**	**RDV**	**References**
**NUTRIENT-RICH COMPONENTS**		
Protein, g^a^^,^^b^	112.5 (m), 90 (w)	NNR (51)
Fiber, g	35 (m), 25 (w)	NNR (51)
Vitamin A, μg RE	750 (m), 650 (w)	EFSA (38)
Vitamin C, mg	110 (m), 95 (w)	EFSA (40)
Vitamin E, mg	13 (m), 11 (w)	EFSA (45)
Calcium, mg	1,200	HCNL (52)
Iron, mg	11	EFSA (46)
Magnesium, mg	350 (m), 300 (w)	EFSA (37)
Potassium, mg	3,500	EFSA (48)
Vitamin D, μg	20	HCNL/NNR (51, 52)
Folate, μg DFE	330	EFSA (41)
Vitamin B12, μg	2.8	HCNL (52)
Zinc, mg^c^	11.7 (m), 9.3 (w)	EFSA (42)
Selenium, μg	70	EFSA (43)
Iodine, μg	150	EFSA (44)
Copper, mg	1.6 (m), 1.3 (w)	EFSA (47)
Vitamin B2, mg	1.6	EFSA (50)
PUFA, g^b^^,^^d^	22.2 (m), 17.8 (w)	NNR (51)
Vitamin B1, mg^b^^,^^e^	1.0 (m), 0.8 (w)	EFSA (49)
Vitamin B6, mg	1.7 (m), 1.6 (w)	EFSA (39)
**NUTRIENTS TO LIMIT**		
Saturated fat, g	20	EFSA (53)
Sugar, g	90	EFSA (53)
Sodium, mg^f^	2,400	EFSA (53)

*Population Reference Intakes and Adequate Intakes as set by the European Food Safety Authority (EFSA) (37–50), the Nordic Council of Ministers (NNR) (51), the Health Council of the Netherlands (HCNL) (52) as well as the labelling Reference Intake values as set by the European Food Safety Authority (53) were used as Reference Daily Values (RDV) m, men; w, women*.

a *Values equal to18 energy percent*.

b *Based on EFSA reference intakes of 2,500 kcal and 2,000 kcal reference intakes for men and women, respectively*.

c *EFSA references for mixed diets, containing 600 mg of phytate*.

d *Values equal to 8 energy percent*.

e *Value equal to 0.4 mg per 1,000 kcal*.

f *Value derived from salt reference value using a conversion factor of 2.5*.

The calculation of the E-NRF score comprised several steps, similar to calculating the NRF9.3 (32). First, the scores were calculated for each food item per 100 kcal. Subsequently, these food scores were converted into individual scores by multiplying the amount of energy consumed of each item, in 100-kcal units, by the nutrients to encourage (nutrient-rich; NR) scores and then summing these scores for each subject. Next, the NR index scores were divided by the number of 100-kcal units of the subjects' total energy intake to provide a “weighted average” score. For the nutrients to limit (LIM) score, the same approach was used.

The algorithms used to calculate the E-NRF scores are listed in Table 3 and are based on sums of nutrients where all nutrients were equally weighted (23). The algorithms which combined positive nutrients and nutrients to limit were based on subtracting the negative from the positive sub score (23). Moreover, the scores were calculated per 100 kcal, since this led to the highest percentage of variance accounted for in previous validation studies (54). Higher E-NRF scores indicate higher nutrient density per 100 kcal.

**Table 3:** Algorithms used to calculate the E-NRF index scores.

**Model**	**Algorithm**	**Comment**
NR*n*_100kcal_	∑_i_ = 1–*n* (Nutrient_i_/RDV_i_) * 100	Nutrient_i_ = content of nutrient i in 100-kcal edible portion; RDV_i_ = recommended daily values for nutrient i
LIM3_100kcal_	∑_i_ = 1–3 (Nutrient_i_/MDV_i_) * 100	Nutrient_i_ = content of limiting nutrient i in 100-kcal edible portion; MDV_i_ = recommended daily values for nutrient i
NRF*n.3*_100kcal_	NR*n*–LIM3	Difference between sums

*NRn, nutrient-rich score consisting of n beneficial nutrients dependent on the evaluated E-NRF score; LIM3, limited nutrient score consisting of three nutrients to limit; NRF, nutrient-rich foods score*.

### NU-AGE Index

The E-NRF scores were evaluated against the NU-AGE index. The NU-AGE index is an a priori dietary index developed by Berendsen et al. (55) The NU-AGE index is meant to reflect adherence to guidance based on DRVs and food based dietary guidelines for elderly individuals from Italy (56), the UK (57), the Netherlands (58–62), Poland (63), and France (64), on the modified MyPyramid for Older Adults (65, 66), and nutrient requirements from the European Community (67), and from the Institute of Medicine (68). These recommendations were jointly integrated into NU-AGE Food Based Dietary Guidelines (Table 4), including recommendations on consumption of whole meal bread and wholegrain pasta or rice, fruits, vegetables, legumes, low-fat dairy, low-fat cheese, fish, low-fat meat, and poultry, nuts, eggs, olive oil, fluid and use of a vitamin D supplement, alcohol, salt (sodium), and sweets. The NU-AGE index is a continuous score with 16 components based on adherence to the aforementioned guidelines. For all components a maximum of 10 points can be assigned resulting in a score of 0–160.

**Table 4:** Components of the NU-AGE index and their cut-off (maximum score) and threshold (minimum score) values (55).

**Component**	**Servings**	**Scoring**			
		**Minimum score (0)**	**Lower range^**b**^ (1-10)**	**Maximum score (10)**	**Upper range^**c**^ (10-1)**
Whole meal bread and wholegrain pasta or rice^a^	Bread 4–6 servings/day (140–210 g/day) Pasta/rice 2 ×80 g/week (23 g/day)	Max	1–163 g	163–233 g	233-max
Fruits	2 servings/day (240 g/day)	0 g	0–240 g	≥240 g	
Vegetables	300 g/day	0 g	0–300 g	≥300 g	
Legumes	200 g/week (29 g/day)	0 g	0–29 g	≥29 g	
Low-fat dairy	500 ml/day	0 g	0–500 g	≥500 g	
Low-fat cheese	30 g/day	0 g	0–30 g	≥30 g	
Fish	2 times 125 g/week (36 g/day)	0 g	0–36 g	≥36 g	
Low-fat meat and poultry^a^	4 times 125 g/week (71 g/day)	Max	0–71 g	71–125 g	125-max
Nuts	2 times 20 g/week (6 g/day)	0 g	0–6 g	>6 g	
Eggs	2–4 eggs/week (14–28 g/day)	0 g	0–14 g	>14 g	
Olive oil	20 g/day	0 ml	0–20 ml	≥20 ml	
Fluid	1,500 ml/day	<1,000 ml	1,000–1,500 ml	>1,500 ml	
vitamin D	Use supplement (10 μg/day)	No		Yes	
Alcohol	Max 2 servings/day for men and 1 serving/day for women	>10 g for women >20 g for men		≤ 10 g for women ≤ 20 g for men	
Salt^a^	5 g/day (2,000 mg/day sodium)	≥85th	0–1,500 mg	1,500–2,000mg	2,000–85th
Sweets^a^	Limited use	≥85th		0	0–85th

*Fruit: maximum 1 glass of fresh fruit juices (120 ml) can be counted as one portion of fruit. Nuts: includes salted and unsalted nuts*.

a *The cut-off value at which a participant would score 0 points was based on the 85 or100th (max) percentile (pct) of the data-specific intake distribution as higher intakes are not necessarily better (100th pct wholegrains (g): 696 DNFCS, 343 NU-AGE; 100th pct meat and poultry (g): 261 DNFCS, 110 NU-AGE; 85th pct sodium (mg); 3141 DNFCS, 2920 NU-AGE; 85th pct sweets (g): 195 DNFCS, 130 NU-AGE)*.

b *The range was divided into 10 and then points were given in proportion to the distance from the 0 point cut-off*.

c Calculation of points for dietary intake between the upper limit and the standard intake for maximum number of points: 10—(intake—recommendation upper limit) ^*^10/standard upper limit.

### Statistical Analyses

All statistical analyses were performed with SPSS version 23.0. General characteristics are expressed as mean ± SD or number (percentage) and differences between men and women were tested with independent *t*-tests or Mann Whitney-*U*-test for continuous variables or chi-square test for categorical variables. Spearman correlation coefficients between all E-NRF scores and the NU-AGE index were calculated. Regression analyses were conducted using the NU-AGE index as the dependent variable and the E-NRF scores as independent variable, testing one E-NRF score at a time. The proportion of explained variance (score R^2^ and model R^2^) and standardized regression coefficients (STB) were estimated while adjusting for age and sex by using the following equation:

NU-AGE index = β_0_ + β_1_^*^E-NRF score + β_2_^*^ age in years + β_3_^*^gender

Sensitivity analyses were performed to assess the robustness of the results. The regression analyses were conducted separately for men and women, for lower weight and higher weight subjects (median-split BMI ≤ 27.1 and > 27.1 kg/m^2^ in DNFCS and BMI ≤ 26.1 and > 26.1 kg/m^2^ in NU-AGE), and across levels of energy intake (median-split ≤ 1,937 kcal vs. >1,937 kcal in DNFCS and ≤ 1,844 kcal vs. >1,844 kcal in NU-AGE).

The E-NRF score with the highest proportion of explained variance in both the DNFCS and NU-AGE study was used to score all foods. To provide insight into the nutrient density of food groups, mean index scores per 100 kcal on a food-group level were calculated. Additionally, to study the contribution of food groups, taking into account the amount consumed, mean contribution (percent) of food groups to the individual weighted scores were calculated.

## Results

The DNFCS and NU-AGE study populations had a mean age of 77.1 ± 5.2 and 71.0 ± 4.0 years, a BMI of 27.4 ± 3.8 and 26.1 ± 3.6 kg/m^2^, consisted of 369 (50%) and 111 (44%) men, respectively and the majority did not smoke (90 and 97%, Table 5). Within the DNFCS there was a higher proportion of people with diabetes mellitus (12%) compared to the NU-AGE population (3.6%). Overall, women had higher NU-AGE scores compared to men (64.4 ± 14.2 vs. 60.2 ± 15.0 within DNFCS and 74.2 ± 15.9 vs. 65.1 ± 4.9 within NU-AGE).

**Table 5:** Baseline characteristics of the participants of the DNFCS (*n* = 735) and NU-AGE study (*n* = 250).

	**DNFCS**			**NU-AGE**		
**Characteristic**	**Total population (*n* = 735)**	**Men (*n* = 369)**	**Women (*n* = 366)**	**Total population (*n* = 250)**	**Men (*n* = 111)**	**Women (*n* = 139)**
Age, years	77.1 ± 5.2	76.7 ± 5.0	77.6 ± 5.4^*^	71.0 ± 4.0	70.9 ± 4.2	71.1 ± 3.9^*^
BMI, kg/m^2a^	27.4 ± 3.8	27.2 ± 3.2	27.6 ± 4.3	26.1± 3.6	26.7 ± 3.5	25.5 ± 3.6^*^
Highly educated^b^	166 (22.6)	104 (28.3)	62 (16.9)^*^	35 (14.0)	22 (19.8)	13 (9.4)
**SMOKING STATUS**						
Never	253 (34.4)	55 (14.9)	198 (54.1)^*^	125 (50)	42 (37.8)	83 (59.7)^*^
Former	407 (55.4)	269 (72.9)	138 (37.7)	117 (46.8)	65 (58.6)	52 (37.4)
Current	75 (10.2)	45 (12.2)	30 (8.2)	8 (3.2)	4 (3.6)	4 (2.9)
Physically active^c^	576 (78.4)	280 (75.9)	296 (81.1)	137.8 ± 53.3	141.2 ± 54.1	135 ± 52.7
Diabetes mellitus^d^	88 (12.0)	51 (13.9)	37 (10.1)	9 (3.6)	2 (1.8)	7 (5.0)
Hypertension^d^	218 (29.7)	91 (24.7)	127 (34.7)^*^	83 (33.2)	38 (34.2)	45 (32.4)
Osteoporosis^d^	60 (8.2)	2 (0.5)	58 (15.8)^*^	26 (10.4)	3 (2.7)	23 (16.5)^*^
Energy, kcal	1,964 ± 457	2,164 ± 420	1,763 ± 402^*^	1,901 ± 396	2,086 ± 440	1,754 ± 282^*^
Sodium, mg	2,318 ± 804	2,563 ± 841	2,070 ± 683^*^	2,362 ± 634	2,651 ± 690	2,131 ± 474^*^
Alcohol, g	11.4 ± 16.1	16.0 ± 19.0	6.7 ± 10.6^*^	12.8 ± 11.9	16.4 ± 13.6	9.9 ± 9.4^*^
Fat, EN%	34.6 ± 5.8	34.4 ± 5.6	34.8 ± 6.0	34.3 ± 5.1^*^	33.5 ± 4.5	34.9 ± 5.4^*^
Carbohydrates, EN%	43.4 ± 6.7	42.7 ± 6.9	44.1 ± 6.5^*^	42.1± 6	42.3 ± 5.7	42.0 ± 6.2^*^
Protein, EN%	15.7 ± 3.0	15.4 ± 2.8	15.9 ± 3.1^*^	16.2 ± 2.4	15.9 ± 2.2	16.4 ± 2.5
NU-AGE index	62.4 ± 14.8	60.2 ± 15.0	64.6 ± 14.2	70.2 ± 16.1	65.1 ± 14.9	74.2 ± 15.9^*^

*Values are presented as mean ± SD and number (%)*.

BMI, body mass index; EN%, energy percentage; DNFCS, Dutch National Food Consumption Survey.

a *DNFCS: n = 706 (10 men, 19 women missing)*.

b *Highly educated is defined as higher vocational education or university in DNFCS and as >16 years of education (at least Bachelor degree) in NU-AGE*.

c *Physically active is defined as a minimum of 30 minutes of moderately intense activity ≥5 days a week in DNFCS, and as PASE score within NU-AGE (DNFCS: n = 734; NU-AGE: n = 248)*.

d *DNFCS: n = 734 (1 man missing)*.

* *statistically significant difference between men and women*.

**Figure 1:**
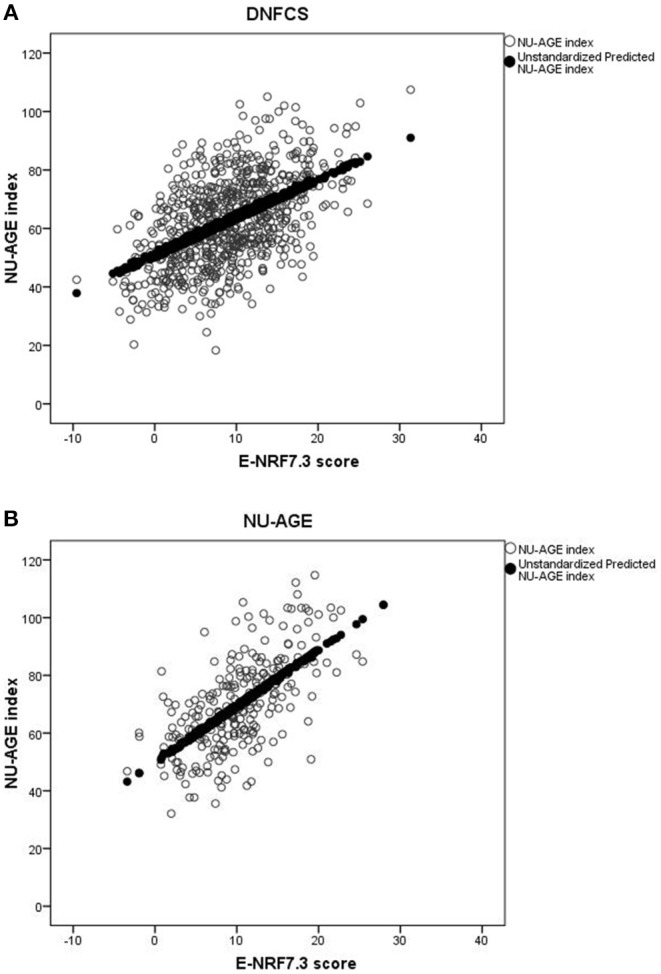
Actual and predicted E-NRF7.3 scores and NU-AGE index values in DNFCS **(A)** and NU-AGE **(B)** populations.

Results of the correlation coefficients and linear regression analyses of the evaluated E-NRF scores on the NU-AGE index showed no large differences between the 27 tested E-NRF scores (Table 6). The LIM3 was inversely correlated to and least predictive of the NU-AGE index score (STB = −0.27, ${\text{R}}_{\text{adj}}^{2}$ = 0.09 in DNFCS and STB = −0.29, ${\text{R}}_{\text{adj}}^{2}$ = 0.05 in NU-AGE).The E-NRF scores combining nutrients to encourage and nutrients to limit were most predictive of the NU-AGE index. Compared to the E-NRF5.3 score (${\text{R}}_{\text{adj}}^{2}$ = 0.22 and 0.36 in DNFCS and NU-AGE, respectively), the nutrients that most improved the prediction of the NU-AGE index in both datasets were magnesium and potassium (${\text{R}}_{\text{adj}}^{2}$ = 0.25 and 0.24 within DNFCS and ${\text{R}}_{\text{adj}}^{2}$ = 0.40 and 0.38 within NU-AGE). Adding PUFA to the E-NRF7.3 score did not substantially improve the prediction (${\text{R}}_{\text{adj}}^{2}$ = 0.26 in DNFCS and 0.42 in NU-AGE). The E-NRF7.3 score showed best prediction in both datasets with ${\text{R}}_{\text{adj}}^{2}$ = 0.27 and 0.41 in DNFCS and NU-AGE. The correlation coefficient between E-NRF7.3 score and the NU-AGE index was 0.49 in DNFCS and 0.64 in NU-AGE. Actual and predicted E-NRF7.3 scores and NU-AGE index values have been graphically presented in Figure 1.

**Table 6:** Correlation coefficients and linear regression analyses of the E-NRF scores on the NU-AGE index within DNFCS (*n* = 735) and NU-AGE study (*n* = 250).

	**DNFCS (*****n*** **=** **735)**							**NU-AGE NL (*****n*** **=** **250)**						
**Model**	**Mean**	**SD**	**Spearman's ρ**	**Linear regression with NU-AGE index**^**a**^				**Mean**	**SD**	**Spearman's ρ**	**Linear regression with NU-AGE index**^**a**^			
				**β**	**STB**	**R^**2**^ score**	**R^**2**^ model**				**β**	**STB**	**R^**2**^ score**	**R^**2**^ model**
LIM3	18.0	2.3	−0.24	−1.76	−0.27	0.05	0.09	17.5	1.9	−0.24	−2.52	−0.29	0.05	0.15
E-NR5	17.5	3.8	0.42	1.72	0.44	0.19	0.19	18.0	3.2	0.59	2.95	0.59	0.34	0.34
E-NR6-Zn	22.5	4.8	0.41	1.36	0.44	0.17	0.17	23.1	4.1	0.57	2.25	0.57	0.30	0.30
E-NR6-I	22.8	4.6	0.41	1.37	0.43	0.18	0.18	23.6	3.8	0.54	2.11	0.50	0.28	0.28
E-NR6-VitE	23.0	4.9	0.42	1.34	0.44	0.18	0.18	23.3	4.4	0.57	2.01	0.55	0.30	0.30
E-NR6-VitC	22.2	5.4	0.44	1.27	0.46	0.20	0.20	22.8	4.9	0.58	1.95	0.59	0.34	0.33
E-NR6-Se	20.7	4.2	0.44	1.63	0.47	0.21	0.21	21.4	3.6	0.56	2.46	0.55	0.32	0.31
E-NR6-B12	25.7	5.8	0.41	0.95	0.37	0.15	0.15	26.1	4.7	0.46	1.41	0.41	0.21	0.22
E-NR6-B6	23.0	5.2	0.36	0.95	0.34	0.12	0.12	22.9	4.2	0.51	1.83	0.48	0.26	0.26
E-NR6-Fe	22.2	4.4	0.44	1.55	0.46	0.20	0.20	23.1	3.7	0.59	2.52	0.58	0.33	0.33
E-NR6-Mg	22.4	4.5	0.46	1.65	0.51	0.23	0.23	23.2	4.0	0.64	2.68	0.67	0.39	0.39
E-NR6-K	22.1	4.4	0.44	1.58	0.47	0.21	0.21	22.7	3.8	0.60	2.59	0.61	0.37	0.36
E-NR7	27.0	5.2	0.47	1.49	0.52	0.24	0.24	28.0	4.7	0.64	2.32	0.67	0.40	0.40
E-NR8	30.7	5.6	0.47	1.42	0.53	0.24	0.25	31.9	5.2	0.65	2.24	0.72	0.42	0.42
E-NRF5.3	−0.5	4.6	0.45	1.48	0.46	0.22	0.22	0.4	3.9	0.61	2.35	0.57	0.36	0.36
E-NRF6.3-Zn	4.5	5.5	0.44	1.26	0.46	0.21	0.21	5.6	4.7	0.60	1.95	0.56	0.33	0.33
E-NRF6.3-I	4.8	5.4	0.44	1.26	0.46	0.22	0.22	6.0	4.5	0.57	1.84	0.52	0.30	0.32
E-NRF6.3-VitE	5.0	5.7	0.44	1.17	0.45	0.21	0.21	5.8	5.0	0.60	1.76	0.55	0.33	0.33
E-NRF6.3-VitC	4.2	6.0	0.48	1.21	0.49	0.24	0.24	5.2	5.3	0.61	1.83	0.60	0.38	0.38
E-NRF6.3-Se	2.7	5.1	0.46	1.40	0.48	0.24	0.24	3.8	4.3	0.59	2.02	0.54	0.33	0.33
E-NRF6.3-B12	7.6	6.4	0.44	1.00	0.43	0.19	0.20	8.5	5.2	0.50	1.41	0.46	0.25	0.26
E-NRF6.3-B6	5.0	6.0	0.39	0.93	0.38	0.15	0.16	5.4	4.9	0.54	1.62	0.49	0.28	0.29
E-NRF6.3-Fe	4.2	5.2	0.46	1.34	0.47	0.23	0.23	5.5	4.4	0.60	2.05	0.56	0.35	0.35
E-NRF6.3-Mg	4.4	5.3	0.47	1.41	0.51	0.25	0.25	5.7	4.7	0.65	2.14	0.62	0.40	0.40
E-NRF6.3-K	4.1	5.2	0.47	1.39	0.49	0.24	0.24	5.2	4.5	0.62	2.12	0.59	0.38	0.38
E-NRF7.3	8.9	5.9	0.49	1.31	0.52	0.27	0.27	10.4	5.3	0.64	1.92	0.63	0.41	0.41
E-NRF8.3	12.7	6.4	0.49	1.20	0.52	0.26	0.26	14.3	5.8	0.66	1.82	0.66	0.42	0.42

*E-NRF, Elderly nutrient-rich foods score; E-NR, Elderly nutrient-rich score; LIM, nutrients to limit; STB, standardized regression coefficient*.

a *Models adjusted for age and sex*.

In both study populations, women had a higher mean E-NRF7.3 score compared to men (10.7 ± 6.0 vs. 7.2 ± 5.3 within DNFCS and 12.3 ± 5.1 vs. 8.0 ± 4.6 within NU-AGE). Within the DNFCS, the E-NRF7.3 score predicted the NU-AGE index similarly for men and women (${\text{R}}_{\text{adj}}^{2}$ = 0.26 and 0.24, respectively). Within NU-AGE, the prediction was stronger in women compared to men (${\text{R}}_{\text{adj}}^{2}$ = 0.43 vs. ${\text{R}}_{\text{adj}}^{2}$ = 0.25, Supplementary Table 1).

Within the DNFCS the mean E-NRF7.3 score was lower in subjects with a lower BMI compared to subjects with a higher BMI (mean E-NRF7.3 score 8.4 ± 6.1 vs. 9.6 ± 5.6) whereas the NU-AGE score was comparable between the two groups (62.7 ± 16.3 vs. 62.3 ± 13.3, Supplementary Table 2). Within the NU-AGE population the mean E-NRF7.3 score was also comparable between the two BMI groups (10.5 ± 5.8 vs. 10.4 ± 4.8), however, the mean NU-AGE score was substantially higher in those with a lower BMI compared to those with a higher BMI (73.6 ± 17.0 vs. 66.6 ± 14.4). The prediction was consistently higher among subjects with a lower BMI compared to those with a higher BMI within the DNFCS (${\text{R}}_{\text{adj}}^{2}$ = 0.30 vs. ${\text{R}}_{\text{adj}}^{2}$ = 0.24) and also within the NU-AGE study (${\text{R}}_{\text{adj}}^{2}$ = 0.45 vs. ${\text{R}}_{\text{adj}}^{2}$ = 0.39).

**Figure 2:**
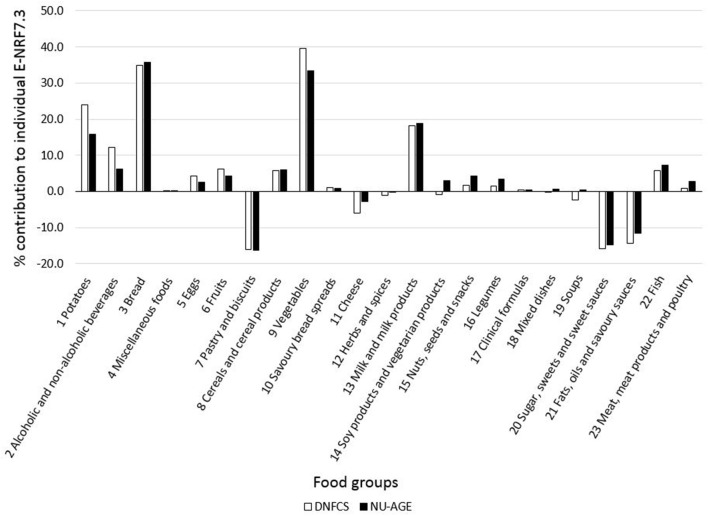
Mean contribution (%) of food groups to individual E-NRF7.3 scores in both DNFCS (*n* = 735) and NU-AGE (*n* = 250).

Food groups that had the highest E-NRF7.3 score on food-item level in both study populations were vegetables, legumes and fish, making up three of the top four in both (Table 7). Clinical formulae scored second highest in NU-AGE, whereas in DNFCS miscellaneous foods were the third highest. Food items that had lowest E-NRF7.3 scores were herbs and spices, soups, sugar, sweets and sweet sauces and pastry and biscuits in both populations. With respect to individual E-NRF7.3 scores, taking into account the choice of food items and the amount consumed, vegetables (40 and 33%), bread (35 and 36%), potatoes (24 and 16%) and milk and milk products (18 and 19%) contributed most to individual E-NRF7.3 scores in DNFCS and NU-AGE, respectively (Figure 2). However, inter-individual variation was quite high (Supplementary Table 3).

**Table 7:** Mean E-NRF index scores per food group, calculated per 100 kcal of foods consumed within DNFCS (*n* = 735) and NU-AGE (*n* = 250).

	**DNFCS**							**NU-AGE**						
		**LIM3**		**E-NR7**		**E-NRF7.3**			**LIM3**		**E-NR7**		**E-NRF7.3**	
**Food groups**	**Number of foods**	**Mean**	**SD**	**Mean**	**SD**	**Mean**	**SD**	**Number of foods**	**Mean**	**SD**	**Mean**	**SD**	**Mean**	**SD**
1 Potatoes	12	9.7	6.4	26.8	9.3	17.1	11.9	20	7.7	6.4	26.3	12.6	18.6	14.8
2 Alcoholic and non-alcoholic beverages	140	23.4	16.5	21.1	37.2	−2.3	32.5	89	21.6	19.2	21.8	42.2	0.2	37.0
3 Bread	83	11.4	3.6	22.4	8.6	11.0	10.1	75	11.5	3.7	21.7	9.4	10.1	10.6
4 Miscellaneous foods	15	26.9	17.0	67.4	66.5	40.5	66.9	19	18.5	19.9	43.4	63.2	24.9	55.0
5 Eggs	2	15.5	0.6	39.2	1.3	23.7	0.7	7	15.8	0.4	31.8	9.4	16.0	9.5
6 Fruits	73	22.6	9.4	34.7	22.8	12.1	26.9	74	22.2	9.4	35.5	22.3	13.3	26.3
7 Pastry and biscuits	126	20.3	5.1	10.3	4.6	−9.9	8.6	91	20.0	5.5	10.2	5.6	−9.8	9.0
8 Cereals and cereal products	41	5.7	5.3	30.5	25.7	24.8	25.0	43	4.0	4.3	25.7	25.1	21.6	24.0
9 Vegetables	142	21.0	22.4	136.2	78.6	115.2	83.3	130	20.9	23.1	130.7	66.3	109.8	72.9
10 Savory bread spreads	6	14.6	8.2	21.9	9.9	7.3	15.4	6	14.6	8.2	21.9	9.9	7.3	15.4
11 Cheese	47	35.8	5.3	30.4	11.9	−5.4	14.1	48	35.4	6.3	29.4	11.8	−6.0	14.5
12 Herbs and spices	17	69.7	39.4	30.5	22.1	−39.2	49.0	11	55.3	44.2	42.3	31.1	−13.0	60.9
13 Milk and milk products	124	24.5	4.8	31.5	19.4	6.9	22.3	102	24.0	4.7	32.3	19.9	8.2	22.8
14 Soy products and vegetarian products	22	29.7	37.0	52.1	29.7	22.4	54.0	23	25.5	32.0	50.6	25.5	25.1	49.3
15 Nuts, seeds and snacks	59	13.2	6.8	18.2	10.8	5.0	15.4	60	14.0	7.5	17.3	10.9	3.3	16.1
16 Legumes	8	9.2	8.9	57.6	6.2	48.4	13.4	8	5.9	9.1	64.2	8.1	58.3	15.2
17 Clinical formulas	9	13.9	7.4	38.4	22.6	24.6	23.9	5	11.5	5.7	70.6	26.9	59.1	24.5
18 Mixed dishes	4	14.4	9.5	16.1	5.9	1.7	7.0	38	16.8	8.4	21.5	13.7	4.7	14.4
19 Soups	20	63.7	30.5	44.2	29.6	−19.5	34.1	21	51.3	30.4	41.0	29.0	−10.3	30.7
20 Sugar, sweets and sweet sauces	77	23.0	6.4	8.6	6.7	−14.4	9.7	67	22.8	6.9	9.2	7.4	−13.6	9.2
21 Fats, oils and savory sauces	145	21.4	17.3	13.3	22.4	−8.0	28.8	88	22.5	20.4	12.8	21.4	−9.7	29.6
22 Fish	39	15.3	10.7	44.8	16.4	29.5	21.9	57	17.2	16.3	42.9	19.4	25.7	27.1
23 Meat, meat products and poultry	136	21.3	13.1	28.2	22.4	6.9	27.6	157	19.7	12.3	26.4	21.0	6.6	26.2

*E-NR, Elderly nutrient-rich score; LIM, nutrients to limit; E-NRF, Elderly nutrient-rich foods score*.

## Discussion

This study aimed to develop and evaluate a nutrient-rich food score specifically capturing dietary reference values for the older population. We have evaluated multiple combinations of nutrients in relation to an index for a healthful diet specifically for elderly people, the NU-AGE index. The score that best predicted the NU-AGE index in both the DNFCS and NU-AGE study populations included seven nutrients to encourage and 3 nutrients to limit, on a 100-kcal basis, the E-NRF7.3 score, with a model ${\text{R}}_{\text{adj}}^{2}$ of 0.27 in DNFCS and 0.41 in NU-AGE. The E-NRF7.3 score performed well in both men and women and in normal-weight and overweight participants.

To our knowledge this is the first study specifically developing a nutrient-rich food score for the aging population. It has previously been discussed that the NRF9.3 could be of limited use within a population of elderly people, as the NRF9.3 is based on recommended daily allowances for a US adult population instead of for an older population (17). As a result, the NRF9.3 does not specifically contain nutrients that are relevant for the older population segment. Therefore, we based our choice of qualifying nutrients on shortfall nutrients, on nutrients that have been shown to be of inadequate intake, and nutrients that were related to relevant health outcomes, specifically within European older populations, resulting in a selection of protein, dietary fiber, vitamin D, folate, calcium, and magnesium as nutrients to encourage and saturated fat, sodium, and total mono- and disaccharides as nutrients to limit.

Of the tested nutrient combinations, the E-NRF7.3 score performed best with an ${\text{R}}_{\text{adj}}^{2}$ of 0.27 in the DNFCS and 0.41 in the NU-AGE study. If there had been a perfect fit between the E-NRF and NU-AGE index, a correlation of R^2^ = 1.0 would be observed. Participants with a higher E-NRF score can be considered to have a healthier dietary pattern compared to those with a lower E-NRF score. The NRF9.3 has previously been validated against the Healthy Eating Index (HEI) 2005 and the DHD index. The proportion of explained variance of the E-NRF score against the NU-AGE index was slightly lower compared with Fulgoni et al., reporting an ${\text{R}}_{\text{adj}}^{2}$ of 0.45 between the HEI-2005 and the NRF9.3 (23), while it was somewhat higher compared with Sluik et al. who reported an ${\text{R}}_{\text{adj}}^{2}$ of 0.34 between the DHD index and the NRF9.3 (24). Overall, the ${\text{R}}_{\text{adj}}^{2}$ was consistently higher in the NU-AGE study population than in the DNFCS. This might be caused by differences between the study populations. Specifically, the NU-AGE population was younger, had a lower BMI, a lower prevalence of diabetes, and contained fewer smokers compared to the DNFCS. Adding more vitamins and minerals to the model did not necessarily improve the prediction (${\text{R}}_{\text{adj}}^{2}$ changed from 0.27 for the E-NRF7.3 score to 0.26 for the E-NRF8.3 score in the DNFCS and from 0.41 to 0.42 in the NU-AGE study). Adding some vitamins even lowered the prediction as is visible in the lowest R^2^ of 0.16 for E-NRF6.3B6 in DNFCS and of 0.26 for E-NRF6.3B12 score in NU-AGE. This was also demonstrated by the selection of selenium as this mineral has previously been shown to be a shortfall nutrient (14, 17). However, adding this mineral to the E-NRF score resulted in a better ${\text{R}}_{\text{adj}}^{2}$ in neither the DNFCS, nor in the NU-AGE study population. As such, it was decided to select the best model ${\text{R}}_{\text{adj}}^{2}$ with the least nutrients to increase the practical applicability of the E-NRF score.

Within the NU-AGE population, the prediction of the NU-AGE index was higher in women than in men, but not in DNFCS. This could be a result of a higher NU-AGE score and a lower BMI of the women compared to men in NU-AGE. In DNFCS these characteristics did not show large differences.

Moreover, the prediction models were consistently better in the NU-AGE population (${\text{R}}_{\text{adj}}^{2}$ E-NRF7.3 score = 0.41) compared to the DNFCS (${\text{R}}_{\text{adj}}^{2}$ E-NRF7.3 score = 0.27). A possible explanation could be the difference in dietary assessment methods, as well as number of days on which dietary intake was assessed. The DNFCS used two-day 24-h recalls, whereas the NU-AGE study used 7-day food records. The two populations also differed in age range (65–79 and 70–94 years of age in the NU-AGE and DNFCS, respectively), which could contribute to differences in reported dietary intake as a result of possible memory complaints, or true differences in dietary intakes as a result of advancing age.

In both study populations, food groups with the highest E-NRF7.3 score were vegetables, legumes and fish. This is partly in line with previous studies in which vegetables, legumes and fruits were observed to have the highest NRF9.3 scores (24, 69). The absence of fruit and presence of fish among the highest scoring food groups in our study compared to NRF9.3 scores in previous studies could be the result of the lack of vitamins present in fruit and the relative importance of protein and vitamin D in fish in the E-NRF7.3 score. An additional difference compared to previous studies using the NRF9.3 is the high E-NRF7.3 score of miscellaneous foods and clinical formulae. Studies using the NRF9.3 used different definitions of food groups or lacked these groups altogether (24, 69) which limits comparability. Considering the various miscellaneous foods such as coconut, cacao, seaweed and the high nutrient density of clinical formulae, which nearly all contain added vitamins and minerals (70) the observed high E-NRF7.3 scores can be explained. The practical relevance of the high E-NRF7.3 scores for these two food groups in our populations is limited as their contributions to the total individual E-NRF7.3 score are very small (<0.5%).

Food groups that had the largest contribution to the individual E-NRF scores within the two study populations were vegetables, bread, potatoes and milk and milk products, similar to the main contributors to the NRF9.3 in another Dutch cohort (24). These food groups are different from the food groups with the highest E-NRF score, as individual weighted E-NRF scores not only depend on the E-NRF score on the food item level, but also on which products are eaten in which amount, as previously discussed by Sluik et al. (24).

While calculating a nutrient-rich food score there are several methodological steps and decisions to be taken which will be discussed below. First of all, in the development of the E-NRF7.3 score, we chose a 100-kcal portion basis. It has previously been shown that the performance of the NRF9.3 on a 100 kcal basis is better than scores based on reference amounts customarily consumed (23) and best reflects the ratio of nutrients to calories, the original definition of nutrient density (22). Additionally, as foods and beverages are consumed in largely varying portions (22), expressing the nutrient density independent of serving size is preferred (21).

Secondly, the decision was made to cap the percentage of nutrients at 100% of the DRV, to prevent high single nutrient contents from producing extremely high scores, as was previously observed by others (21, 24). Moreover, Sluik et al., observed that uncapped scores systematically explained less variance of the DHD index (24).

Thirdly, the E-NRF scores were based on DRVs as set by the European Food Safety Authority, the Nordic Nutrient Recommendations, the Health Council of the Netherlands and the labeling reference intake values as set by the European Food Safety Authority (37–53). There are some differences between these values and the DRVs published by the Institute of Medicine as used for the original NRF9.3. However, it has previously been shown that using American instead of European recommendations did not influence the prediction of the DHD index (24). As we aimed to develop a nutrient-rich food score specifically for the aging European population, we have chosen to use DRV's relevant for this population.

Lastly, selecting nutrients and the way of defining them are important. Data on *total* mono- and disaccharides were available, in contrast to *added* mono- and disaccharides. The latter has been shown to be related to the micronutrient density of the diet (71). However, when Sluik et al. tested both *added* and *total* mono- and disaccharides in the NRF9.3, the model with *total* mono- and disaccharides performed best (24). Furthermore, vitamin K was selected as relevant nutrient as it is associated with relevant health outcomes for the elderly population (15). However, no data on vitamin K intake was available as the NEVO table of 2011 did not contain this nutrient. Future studies could evaluate the effect of including vitamin K in the E-NRF7.3 score.

Our study has some limitations. First, the E-NRF7.3 has been correlated with the NU-AGE index only. The NU-AGE index is the first and only index to measure adherence to guidance based on DRVs and food based dietary guidelines for European elderly individuals (55). The NU-AGE index consists of 16 components, including the use of a vitamin D supplement, resulting in a score ranging from 0 to 160 (55). It has been shown to be able to rank participants from the entire NU-AGE study including over 1,250 older adults according to their adherence to the guidelines (55). Furthermore, higher scores for the NU-AGE index were associated with reduced rates of bone loss at the femoral neck in individuals with osteoporosis (72), an improvement of systolic blood pressure, arterial stiffness (73), global cognition, and episodic memory (74). However, the validity of the E-NRF7.3 score should be confirmed in relation to relevant health outcomes, markers of nutritional status and in other study populations as well. Second, the E-NRF7.3 score does not take into account individual differences in nutrient requirements as personalized nutrition is still a relatively new research area. Once the validity of the E-NRF7.3 score has been studied in more depth, the score could be a useful tool to support nutrition and health claims of foods and to educate older populations to identify nutrient-rich foods for better diet quality. At last, for the E-NRF7.3 score to be used as a tool to address malnutrition, the score could be updated to include multiple risk factors that underlie malnutrition, including physical, social, and medical factors (75).

To conclude, we have developed a nutrient-rich food score specifically targeted at measuring nutrient density of foods and quality of diets from European elderly people. The E-NRF7.3 score was able to rank participants according to their adherence to the NU-AGE index. In future, this newly developed E-NRF7.3 score should be validated against relevant health outcomes for the older population, and more objective markers of dietary intake.

## Data Availability

The datasets generated for this study are available on request to the corresponding author.

## Author Contributions

AB and LdG designed the study. CK and AB analyzed the data and interpreted the data and drafted the manuscript. LdG interpreted the data and critically revised the manuscript for important intellectual content.

### Conflict of Interest Statement

The authors declare that the research was conducted in the absence of any commercial or financial relationships that could be construed as a potential conflict of interest.
