# Supplementary material for: The Newly Developed Elderly Nutrient-Rich Food Score Is a Useful Tool to Assess Nutrient Density in European Older Adults
Source: Front Nutr. 2019 Aug 2;6:119. doi: 10.3389/fnut.2019.00119 (PMC6688200; doi:10.3389/fnut.2019.00119)
Supplement: Supplementary file 1 [file Table_1.docx]

**Supplementary tables**

**Supplementary Table 1.** Mean E-NRF score and NU-AGE index and their associations by gender in Dutch National Food Consumption Survey (n=735) and NU-AGE population (n=250)

|  | Men | |  | Women | |  | Linear regression men^a^ | | |  | Linear regression women^a^ | | |
| --- | --- | --- | --- | --- | --- | --- | --- | --- | --- | --- | --- | --- | --- |
| Model | Mean | SD |  | Mean | SD |  | β | STB | R^2^ |  | β | STB | R^2^ |
| **DNFCS** | | | | | | | | | | | | | |
| LIM3 | 17.6 | 2.3 |  | 18.4 | 2.3 |  | -1.69 | -0.25 | 0.06 |  | -1.82 | -0.29 | 0.08 |
| E-NR7 | 24.8 | 4.4 |  | 29.1 | 5.0 |  | 1.71 | 0.50 | 0.24 |  | 1.31 | 0.46 | 0.21 |
| E-NRF7.3 | 7.2 | 5.3 |  | 10.7 | 6.0 |  | 1.48 | 0.52 | 0.26 |  | 1.16 | 0.49 | 0.24 |
| NU-AGE | 60.1 | 15.0 |  | 64.6 | 14.2 |  |  |  |  |  |  |  |  |
| **NU-AGE** | | | | | | | | | | | | | |
| LIM3 | 17.2 | 2.1 |  | 17.8 | 1.6 |  | -1.53 | -0.21 | 0.03 |  | -3.76 | -0.39 | 0.14 |
| E-NR7 | 25.2 | 3.6 |  | 30.1 | 4.3 |  | 2.23 | 0.53 | 0.27 |  | 2.36 | 0.64 | 0.40 |
| E-NRF7.3 | 8.0 | 4.6 |  | 12.3 | 5.1 |  | 1.68 | 0.51 | 0.25 |  | 2.07 | 0.66 | 0.43 |
| NU-AGE | 65.1 | 14.9 |  | 74.2 | 15.9 |  |  |  |  |  |  |  |  |
| E-NR, Elderly nutrient-rich score; E-NRF, Elderly nutrient-rich foods score; LIM, nutrients to limit; STB, standardised regression coefficient  ^a^ Adjusted for age | | | | | | | | | | | | | |

**Supplementary Table 2.** Mean E-NRF indices and NU-AGE index, and their associations by median BMI in Dutch National Food Consumption Survey (n=706) and NU-AGE population (n=250)

|  | BMI ≤ median^b^ | |  | BMI > median^b^ | |  | Linear regression  BMI ≤ median^ab^ | | |  | Linear regression  BMI > median^ab^ | | |
| --- | --- | --- | --- | --- | --- | --- | --- | --- | --- | --- | --- | --- | --- |
| Model | Mean | SD |  | Mean | SD |  | β | STB | R^2^ |  | β | STB | R^2^ |
| **DNFCS** | | | | | | | | | | | | | |
| LIM3 | 18.1 | 2.4 |  | 17.9 | 2.1 |  | -2.40 | -0.36 | 0.14 |  | -1.06 | -0.17 | 0.04 |
| E-NR7 | 26.5 | 5.1 |  | 27.5 | 5.1 |  | 1.69 | 0.53 | 0.25 |  | 1.33 | 0.52 | 0.24 |
| E-NRF7.3 | 8.4 | 6.1 |  | 9.6 | 5.6 |  | 1.48 | 0.55 | 0.30 |  | 1.18 | 0.50 | 0.24 |
| NU-AGE | 62.7 | 16.3 |  | 62.3 | 13.3 |  |  |  |  |  |  |  |  |
| **NU-AGE** | | | | | | | | | | | | | |
| LIM3 | 17.7 | 1.8 |  | 17.4 | 1.9 |  | -3.34 | -0.36 | 0.13 |  | -2.00 | -0.27 | 0.21 |
| E-NR7 | 28.1 | 5.0 |  | 27.8 | 4.3 |  | 2.52 | 0.74 | 0.46 |  | 1.97 | 0.59 | 0.36 |
| E-NRF7.3 | 10.5 | 5.8 |  | 10.4 | 4.8 |  | 2.07 | 0.70 | 0.45 |  | 1.71 | 0.57 | 0.39 |
| NU-AGE | 73.6 | 17.0 |  | 66.6 | 14.4 |  |  |  |  |  |  |  |  |
| E-NR, Elderly nutrient-rich score; E-NRF, Elderly nutrient-rich foods score; LIM, nutrients to limit; STB, standardised regression coefficient  ^a^ Adjusted for age and gender  ^b^ Median BMI: 27.1 kg/m^2^ in DNFCS, 26.1 kg/m^2^ in NU-AGE | | | | | | | | | | | | | |

**Supplementary Table 3.** Mean contribution of food groups to total individual LIM3, E-NR7 and E-NRF7.3, taking into account consumed portions, calculated in the Dutch National Food Consumption Survey (DNFCS, n=735) and NU-AGE population (n=250) separately

|  | **DNFCS** | | | | | | | |  | **NU-AGE** | | | | | | | |
| --- | --- | --- | --- | --- | --- | --- | --- | --- | --- | --- | --- | --- | --- | --- | --- | --- | --- |
|  | LIM3 | |  | E-NR7 | |  | E-NRF7.3 | |  | LIM3 | |  | E-NR7 | |  | E-NRF7.3 | |
| Food groups | Mean | SD |  | Mean | SD |  | Mean | SD |  | Mean | SD |  | Mean | SD |  | Mean | SD |
| 1 Potatoes | 1 | 1 |  | 6 | 4 |  | 24 | 115 |  | 1 | 1 |  | 4 | 3 |  | 16 | 48 |
| 2 Alcoholic and non-alcoholic beverages | 5 | 5 |  | 6 | 4 |  | 12 | 55 |  | 5 | 3 |  | 6 | 4 |  | 6 | 55 |
| 3 Bread | 11 | 4 |  | 16 | 6 |  | 35 | 144 |  | 11 | 4 |  | 17 | 6 |  | 36 | 119 |
| 4 Miscellaneous foods | 0 | 1 |  | 0 | 1 |  | 0 | 4 |  | 0 | 1 |  | 0 | 0 |  | 0 | 2 |
| 5 Eggs | 1 | 1 |  | 1 | 2 |  | 4 | 15 |  | 1 | 1 |  | 2 | 2 |  | 3 | 18 |
| 6 Fruits | 6 | 5 |  | 6 | 5 |  | 6 | 39 |  | 7 | 5 |  | 7 | 5 |  | 4 | 143 |
| 7 Pastry and biscuits | 10 | 7 |  | 3 | 2 |  | -16 | 204 |  | 9 | 6 |  | 3 | 2 |  | -16 | 51 |
| 8 Cereals and cereal products | 1 | 2 |  | 2 | 3 |  | 6 | 29 |  | 1 | 2 |  | 3 | 3 |  | 6 | 19 |
| 9 Vegetables | 2 | 2 |  | 11 | 6 |  | 40 | 187 |  | 2 | 1 |  | 11 | 5 |  | 33 | 99 |
| 10 Savory bread spreads | 0 | 1 |  | 0 | 1 |  | 1 | 9 |  | 1 | 1 |  | 1 | 1 |  | 1 | 2 |
| 11 Cheese | 10 | 8 |  | 7 | 5 |  | -6 | 78 |  | 12 | 7 |  | 7 | 4 |  | -3 | 82 |
| 12 Herbs and spices | 0 | 1 |  | 0 | 0 |  | -1 | 15 |  | 0 | 1 |  | 0 | 0 |  | 0 | 2 |
| 13 Milk and milk products | 15 | 9 |  | 15 | 9 |  | 18 | 84 |  | 12 | 6 |  | 14 | 7 |  | 19 | 39 |
| 14 Soy products and vegetarian products | 1 | 2 |  | 1 | 3 |  | -1 | 30 |  | 1 | 2 |  | 1 | 3 |  | 3 | 34 |
| 15 Nuts, seeds and snacks | 2 | 4 |  | 2 | 4 |  | 2 | 39 |  | 3 | 3 |  | 3 | 3 |  | 4 | 23 |
| 16 Legumes | 0 | 1 |  | 0 | 2 |  | 1 | 11 |  | 0 | 0 |  | 1 | 2 |  | 4 | 29 |
| 17 Clinical formulas | 0 | 1 |  | 0 | 2 |  | 0 | 4 |  | 0 | 1 |  | 0 | 3 |  | 0 | 5 |
| 18 Mixed dishes | 0 | 0 |  | 0 | 1 |  | 0 | 5 |  | 2 | 3 |  | 2 | 3 |  | 1 | 10 |
| 19 Soups | 3 | 5 |  | 2 | 3 |  | -2 | 35 |  | 4 | 4 |  | 3 | 3 |  | 0 | 13 |
| 20 Sugar, sweets and sweet sauces | 8 | 7 |  | 2 | 2 |  | -16 | 196 |  | 7 | 5 |  | 1 | 1 |  | -15 | 46 |
| 21 Fats, oils and savory sauces | 11 | 7 |  | 7 | 6 |  | -14 | 192 |  | 7 | 4 |  | 4 | 5 |  | -12 | 239 |
| 22 Fish | 2 | 4 |  | 3 | 5 |  | 6 | 20 |  | 3 | 3 |  | 4 | 4 |  | 7 | 14 |
| 23 Meat, meat products and poultry | 11 | 8 |  | 9 | 5 |  | 1 | 87 |  | 10 | 6 |  | 7 | 4 |  | 3 | 65 |

E-NR, Elderly nutrient-rich score; E-NRF, Elderly nutrient-rich foods score; LIM, nutrients to limit
